# Factors associated with ART interruption during the COVID-19 crisis in Burundi (the EPIC community-based research program)

**DOI:** 10.1038/s41598-024-63805-2

**Published:** 2024-06-08

**Authors:** Annabelle Niyongabo, Virginie Villes, Rokhaya Diagne, Juliana Castro Avila, Jean-Michel Mutima, Dévote Gakima, Pélagie Nimbona, Evangéline Niyoncuti, Elvis Rwamuco, Martin Manirakiza, Lucas Riegel, Nicolas Lorente, Rosemary M. Delabre, Daniela Rojas Castro

**Affiliations:** 1 Community-based Research Laboratory, Coalition PLUS, Dakar, Sénégal; 2 Association Nationale de Soutien aux Séropositifs et malades du Sida - Santé PLUS (ANSS-Santé PLUS), Bujumbura, Burundi; 3 Community-based Research Laboratory, Coalition PLUS, Pantin, France; 4 Université du Burundi, Bujumbura, Burundi; 5 Centre Estudis Epidemiològics sobre les Infeccions de Transmissió Sexual i Sida de Catalunya (CEEISCAT), Departament de Salut, Generalitat de Catalunya, Badalona, Spain; 6 Centro de Investigación Biomédica en Red de Epidemiología y Salud Pública (CIBERESP), Madrid, Spain; 7 Aix Marseille University, INSERM, IRD, SESSTIM, Sciences Economiques & Sociales de la Santé & Traitement de L’Information Médicale, ISSPAM, Marseille, France

**Keywords:** Diseases, Health care, Risk factors

## Abstract

With a national prevalence of 0.9%, Burundi is close to achieving UNAIDS’ 2025 targets. Despite this, different types of crises periodically disrupt its HIV health services. The community-based program EPIC measured the impact of the COVID-19 health crisis on people living with HIV (PLHIV) in Burundi in 2021. Specifically, it assessed ART interruption and associated factors since the beginning of the pandemic. The study questionnaire was administered to PLHIV in three cities between October and November 2021. Participants were recruited using convenience sampling. Logistic regression models helped identify factors associated with ART interruption. Of the 317 respondents, 37 (11.7%) reported interruption. The majority (79.2%) self-identified as belonging to key populations. Interruption was significantly associated with: fewer HIV medical follow-up visits (adjusted Odds Ratio, aOR = 7.80, p = 0.001) and forced HIV status disclosure (aOR = 4.10, p = 0.004). It was inversely associated with multi-month ART dispensing (aOR = 0.36, p = 0.017) since the beginning of the pandemic and the perception of not having been sufficiently informed by the HIV medical team about the risk of COVID-19 infection (aOR = 0.11, p < 0.001). Our results highlight the importance of multi-month ART dispensing, enhanced communication, and voluntary disclosure of one’s HIV status in preventing ART interruption in times of crises in Burundi.

## Introduction

Antiretroviral treatment (ART) reduces HIV/AIDS morbi-mortality and prevents new HIV infections^1,2^. The UNAIDS’ 2025 targets to reduce the scale of the HIV/AIDS pandemic are as follows: 95% of people living with HIV (PLHIV) will know their serostatus by 2025, 95% of PLHIV aware of their serostatus will be on ART, and 95% of PLHIV on ART will have a suppressed viral load^3^.

Burundi is close to achieving these targets. In 2022, an estimated 82,166 PLHIV were living in the country. HIV prevalence was 0.9% among people aged 15–49 years old^4^, 97% knew their serostatus, and of these 99% had initiated ART; finally, 93% of persons on ART had a suppressed viral load^4^. Despite these promising figures, challenges remain: (i) HIV prevalence is high among key populations: 32.5% among female sex workers (SW)^5^, 15.3% among people who use drugs (PWUD)^6^, and 5.9% among men who have sex with men (MSM)^7^; (ii) the country is periodically affected by political, social, economic, and health crises^8^ which can disrupt HIV health services and hamper the fight against HIV/AIDS^9^; (iii) the fact that little is known about the extent of ART interruption in Burundi – during and outside of crises – may negatively impact achieving UNAIDS’ targets.

The recent global health crisis due to COVID-19 disrupted HIV care systems throughout the world in many ways. For example, activities such as HIV testing^10–12^ and community-based tracing of patients lost to follow-up^11^ were suspended, healthcare workers experienced physical and psychological burnout^13,14^, and the level of privacy during medical consultations was greatly reduced^15^. In some contexts, continuity of HIV care was more resilient. For example, in Uganda, Lebanon and Burundi, home-based and community-based ART dispensing was strengthened and multi-month dispensing of ART was extended to 3 or 6 months^16,17^.

The COVID-19 pandemic was the most recent crisis to hit Burundi. The first wave started in mid-July 2020^18^. Although a full lockdown was not introduced^19^, between January and July 2021 mass gatherings were not allowed and international frontiers were closed^20,21^. This caused mobility restrictions and loss of income^19^, with many businesses closing. The crisis also affected the health system as it led to ART stockouts (especially third-line treatments) and reduced availability of healthcare workers in HIV clinics^11^. In July 2021, the government took compensatory measures by ensuring that community health workers (CHW)^21^ could provide outreach HIV medical care (including ART distribution) to PLHIV in key communities. CHW work in both public health centers and community-based organizations (CBOs). The latter are peer CHW (i.e. they themselves are members of the specific key community). Public health centers provide medical care to half (52.2%) of the PLHIV in Burundi^4^, while CBO provide care to 22.1%^4^.

In January 2020, multi-month dispensing of ART and community-based distribution of ART were introduced in Burundi’s national guidelines as strategies to improve ART adherence through differentiated service delivery^22^. These strategies were implemented on the ground in July 2020^11,17^, when the COVID-19 crisis was officially declared by the government in Burundi and when international organizations such as UNAIDS and PEPFAR highlighted the need for multi-month dispensing of ART strategy in order to reduce frequency of visits to HIV clinics and therefore limit the number of new COVID-19 infections^23,24^.

Using data from the EPIC research program (*Enquêtes pour évaluer l’impact de la crise sanitaire COVID-19 en milieu communautaire*) which was implemented in Burundi by the community-based organization ANSS-Santé PLUS (*Association nationale de soutien aux séropositifs et malades du Sida—Santé PLUS*) in three of its locations between October and November 2021, we aimed to estimate the extent of ART interruption among PLHIV since the beginning of the COVID-19 health crisis in the country, and to assess associated factors, with a view to providing valuable knowledge for ART management during future crises.

## Methods

### Study design

EPIC is a multi-country, cross-sectional, community-based, mixed methods research program initiated in March 2020 (i.e. immediately after the beginning of the COVID-19 pandemic) to assess the impact of the COVID-19 health crisis on people living with or affected by HIV or viral hepatitis, and people working with these populations in community settings. It was implemented by Coalition PLUS^25^, an international union of community-based organizations (CBO) involved in the fight against HIV and viral hepatitis. Founded in 2008, Coalition PLUS unites 16 member organizations and over 100 partner organizations in 52 countries.

EPIC was developed following concerns by community actors’ that PLHIV and key populations were experiencing specific negative consequences of protective measures put in place to limit the COVID-19 pandemic (e.g. restriction of movement, lockdowns) Specifically, the Coalition PLUS research laboratory created a mixed working group including researchers and community actors, to build a common protocol for this multi-country study. In keeping with the key values of community-based research^26^, several Coalition PLUS member and partner organizations actively participated in all the stages of EPIC (determining the research question and aims, developing the data collection tools, study implementation, and data analysis).

EPIC was developed to be customizable at the local level in order to accommodate the diverse themes of interest and target populations, as well as the realities in the field created by the exceptional circumstances of the COVID-19 pandemic, especially in the context of the Coalition PLUS network. More specifically, although there was a common basis for all the organizations implementing the program, data collection tools could be modified depending on the target population(s) and the specific themes of interest. Both of these dimensions were determined by the implementing organizations^27^. In Burundi, the implementation of EPIC was led by ANSS-Santé PLUS which adapted the original EPIC questionnaire in collaboration with academic partners. The questionnaire was translated in Kirundi.

### ANSS-Santé PLUS

ANSS-Santé PLUS is a CBO based in five cities in Burundi (Bujumbura, Gitega, Makamba, Kirundo and Rumonge), which are the capitals of the five homonymous provinces. Since 1993, it has promoted HIV transmission prevention and has improved the well-being of PLHIV and other people affected by HIV. The organization introduced medical and psychosocial care to these groups even before the publication of related recommendations from the Burundi ministry of health. In 1999, ANSS-Santé PLUS negotiated the introduction of generic ART in Burundi with GlaxoSmithKline. The same year, it successfully advocated the establishment of an ART import fund and import tax exemption on ART products. The CBO’s health facility was the first in Burundi to provide integrated care to PLHIV, including medical, psychosocial and therapeutic education through ART adherence support sessions. In 2022, ANSS-Santé PLUS had 6,026 active PLHIV service users, representing 8% of all PLHIV on ART in Burundi^4^. PLHIV who actively use ANSS-Santé PLUS’ services include children, adolescents, adults, pregnant women, and people who self-identify as belonging to one or more key populations (female SW, MSM, trans women, and PWUD).

ANSS-Santé PLUS is also a leader in involving peer CHW in the global care of PLHIV and has a wide network of partner community organizations involved in the prevention and care of HIV. In 2021, it initiated the first national forum promoting community work in the field of HIV care. Furthermore, in 2023, ANSS-Santé PLUS took the lead in writing the national repository of HIV community-based interventions.

As well as community-based work related to HIV care, ANSS-Santé PLUS is involved in HIV prevention among key populations, especially MSM and PWUD. In 2008, the CBO was the first in Burundi to promote the protection of MSM’s human rights. In terms of HIV care, ANSS-Santé PLUS provides differentiated services to PLHIV who self-identify as belonging to key populations and to those who do not.

### Study population

The present analysis focused on quantitative data from EPIC collected among PLHIV in Burundi between October and November 2021 in the premises of ANSS-Santé PLUS in three of the CBO’s locations, specifically the cities of Bujumbura, Gitega and Makamba. This specific period was characterized by the reinforcement of mask wearing policies in public transport following the re-emergence of community-level COVID-19 transmission. In October 2021, the first COVID-19 vaccination campaign was implemented.

Convenience sampling was used to recruit participants as follows: (i) all PLHIV aged 18 years or over who frequented the ANSS-Santé PLUS center in one of the three cities were invited to participate in EPIC; (ii) in order to reach more people, ANSS-Santé PLUS’ PLHIV representatives and peer CHWwho self-identified as MSM, female SW or PWUD, actively promoted participation in the study in their respective networks. It is important to highlight that participants in the study received services either directly from ANSS-Santé PLUS or indirectly through partner organizations of ANSS-Santé PLUS. Data were collected face to face by ANSS-Santé PLUS’ peer CHW who were trained on collecting data for the EPIC questionnaire using Voxco^®^ survey software. This software allows questionnaire data capture even in offline contexts. The questionnaire included four sections: (i) sociodemographics and the general impact of the COVID-19 health crisis, (ii) representations and perceptions of the risk of getting COVID-19, (iii) access to health services (i.e. not only HIV) and prevention tools during the COVID-19 crisis, and (iv) PLHIV clinical characteristics before and during the crisis (HIV diagnosis, ART and barriers to effective treatment during the COVID-19 crisis).

### Outcome of interest

ART interruption was assessed using the question “Have you interrupted your HIV treatment since the beginning of the COVID-19 health crisis?”, with two answer options, Yes or No.

### Explanatory variables

The sociodemographic component of the questionnaire explored the following variables: age, gender self-identity (multiple choices were possible), highest level of education attained, area of residence (urban vs. semi-urban/rural area), province of residence (Bujumbura, Gitega, Makamba), identification with a key population (MSM, PWUD and SW), and the general impact of COVID-19 health crisis on the participant’s life (changes in financial situation, in quality of life, and in quality of sexual, personal and professional lives). The section on being a ‘PLHIV during the COVID-19 crisis’ explored the following variables: changes in medical follow-up, the need for support, being forced to disclose one’s HIV status, and steps taken by the participant since the beginning of the crisis to ensure he/she received ART and to stay informed and connected with friends, family, and his/her community. One questionnaire item focused on the reasons for interrupting ART, with 11 possible answers (multiple choices were possible).

### Statistical analysis

EPIC participants not on ART at the time of data collection were excluded from the present analysis, as were those who did not answer the outcome of interest question. A total of 11 participants were excluded.

Firth’s logistic regression (penalized log-likelihood) was used to reduce small-sample bias (i.e. small arising from the small number of ART interruptions)^28^. To identify factors associated with ART interruption, a univariable analysis was performed. Variables with a p-value < 0.25 in this analysis were considered eligible to enter the multivariable model. A backward selection procedure was used to construct the final multivariable model. Stata/SE 16.0 software (StataCorp LP, College Station, USA) was used for data management and all the analyses.

### Ethical considerations

Ethical approval was obtained from the National Ethics Committee of the National Institute of Public Health (INSP) in accordance with Burundi ethical considerations. Prior to providing signed informed consent and responding to the questionnaire, all respondents were informed that their participation was voluntary, and that their identity and all personal data from the study would remain confidential. They also received information regarding the objective of the study.

## Results

### Description of respondents, N = 317 PLHIV (Table 1)

**Table 1:** Description of socio-demographic and behavioral characteristics of the study sample according to ART interruption (yes/no) since the beginning of the COVID-19 health crisis (N = 317).

	Description, N = 317, n (%)
Age (years) median [IQR]	32 [27–41]
Years on ART median [IQR]	3 [1–6]
Years on ART	
Less than 4 years	185 (58.4)
4 years or more	132 (41.6)
Years on ART	
Before 2020	235 (74.1)
Since 2020	82 (25.8)
Woman	
No	186 (58.7)
Yes	131 (41.3)
Man	
No	190 (59.9)
Yes	127 (40.1)
Trans women	
No	253 (79.8)
Yes	64 (20.2)
Highest level of education	
No schooling/primary education	149 (47.0)
Secondary/higher education	168 (53.0)
Area of residence*	
Urban area	237 (74.8)
Semi urban/rural area	51 (16.1)
Province of residence	
Bujumbura	234 (73.8)
Other (Gitega or Makamba)	83 (26.2)
Self-identifying as an MSM or gay man	
No	173 (54.6)
Yes	144 (45.4)
Self-identifying as a PWUD	
No	246 (77.6)
Yes	71 (22.4)
Self-identifying as an SW	
No	172 (54.3)
Yes	145 (45.7)
Self-identifying as belonging to at least one key population (MSM, PWUD and/or SW)	
No	66 (20.8)
Yes	251 (79.2)
Deteriorated financial situation**	
No	36 (11.4)
Yes	281(88.6)
ANSS-Santé PLUS direct service user***	
No	37 (11.7)
Yes	280 (88.3)
Current quality of life**	
Better/the same	42 (13.3)
Poorer	272 (85.8)
Current sexual quality of life**	
Better/the same	72 (22.7)
Poorer	219 (69.1)
Perceived a very negative impact of the COVID-19 crisis on personal life	
No	206 (64.9)
Yes	107 (33.8)
Perceived a very negative impact of the COVID-19 crisis on professional life	
No	214 (67.5)
Yes	103 (32.5)
Fewer medical follow-up visits****	
No	259 (81.7)
Yes	49 (15.5)
Missed diagnosis/treatment/surgical intervention for an important health issue during the COVID-19 crisis	
No	203 (35.7)
Yes	113 (64.0)
Needed support from friends (excluding PLHIV)****	
Yes, and I received the support needed	87 (27.4)
Yes, but I needed more support	38 (12.0)
Yes, but I didn’t receive any support	90 (28.4)
No, I didn’t need support	91(28.7)
Missing	11 (3.5)
Needed support from a CHW****	
Yes, and I received the support needed	124 (39.1)
Yes, but I needed more support	69 (21.8)
Yes, but I didn’t receive any support	19 (6.0)
No, I didn’t need support	89 (28.1)
Missing	16 (5.0)
Needed support from medical professionals****	
Yes, and I received the support needed	272 (85.8)
Yes, but I needed more support	22 (6.9)
Yes, but I didn’t receive any support	4 (1.3)
No, I didn’t need support	8 (2.5)
Missing	11 (3.5)
Felt sufficiently informed by their HIV medical team about the risk of COVID-19 infection in the context of HIV infection and treatment	
No/do not know	143 (45.1)
Yes	174 (54.9)
Forced to disclose HIV status***	
No	264 (83.3)
Yes	53 (16.7)
Received ART from peer CHW (new government care policy introduced)***	
No	236 (74.5)
Yes	78 ( 24.6)
Received multi-month ART dispensing (new government care policy introduced)***	
No	137 ( 43.22)
Yes	177 ( 55.84)

*Percentages may not sum to 100 because of missing values.

**Compared to before the COVID-19 crisis.

***During the COVID-19 health crisis.

****Since the COVID-19 health crisis started. Missing values were imputed for the most likely category to occur.

A total of 317 PLHIV participated in EPIC in Burundi. Of these, 37 (11.7%) reported ART interruption since the beginning of the COVID-19 health crisis. The majority (73.8%) of participants were from the province of Bujumbura (n = 234), 13.6% came from Gitega (n = 43), and 12.6% from Makamba (n = 40). 74.8% lived in an urban area, and median age was 32 years [IQR: 27–41]. Two-fifths (41.3%) self-identified as women, 40.1% as men, and 20.2% as trans women. Over half reported secondary or higher education (53.0%). The majority (88.3%) were direct service users of ANSS-Santé PLUS. The majority self-identified as belonging to one or more key populations (79.2%). Specifically, 45.7% self-identified as an SW, 45.4% as an MSM, and 22.4% as a PWUD. The percentages of participants declaring a deterioration in their financial situation since the beginning of the COVID-19 health crisis, a poorer quality of life, and a poorer quality of sexual life, were very high (88.6%, 85.8%, and 69.1%, respectively). A third of participants reported that the crisis had had a very negative impact on their personal (33.8%) and professional (32.5%) lives. Moreover, 15.5% reported fewer medical follow-up visits, 16.7% were forced to disclose their HIV status, 24.6% benefited from the changes in ART distribution (i.e. peer CHW providing treatment), and 55.8% reported receiving multi-month ART dispensation during the crisis.

### Factors associated with ART interruption since the beginning of the COVID-19 health crisis in Burundi, N = 317 (Table 2)

**Table 2:** Factors associated with ART interruption in the univariable analysis (N = 317).

	Univariable analysis, N = 317	
	OR [95% CI]	P-value
Age (years) Median [IQR] (per one unit increase)	**0.96 [0.92; 0.99]**	0.029****
Years on ART Median [IQR]	0.98 [0.91; 1.1]	0.724
Years on ART		
Less than 4 years	0.82 [0.41; 1.61]	0.562
4 years or more	1.00	
Years on ART		
Before 2020	0.79 [0.36; 1.79]	0.585
Since 2020	1.00	
Woman		
No	1	
Yes	0.76 [0.36; 1.52]	0.436
Man		
No	1	
Yes	1.03 [0.52; 2.06]	0.518
Trans woman		
No	1	
Yes	1.14 [0.51; 1.52]	0.745
Highest level of education		
No schooling/primary education	**2.24 [1.14; 4.55]**	0.024****
Secondary/higher education	1	
Area of residence		
Urban area	1	
Semi urban/rural area	0.80 [0.31; 2.11]	0.658
Province of residence		
Bujumbura	1	
Other (Gitega or Makamba)	0.66 [0.28; 1.52]	0.328
Self-identifying as an MSM or gay man		
No	1	
Yes	1.31 [0.66; 2.57]	0.440
Self-identifying as a PWUD		
No	1	
Yes	**3.12 [1.54; 6.31]**	0.002****
Self-identifying as an SW		
No	1	
Yes	0.89 [0.45;1.78]	0.757
Self-identifying as belonging to at least one key population (MSM, PWUD and/or SW)		
No	1	
Yes	0.35 [0.11;1.05]	0.068****
Deteriorated financial situation*		
No	1	
Yes	1.98 [0.53; 7.52]	0.312
ANSS-Santé PLUS direct service user**		
No	1	0.039****
Yes	**0.41 [0.17; 0.95]**	
Current quality of life*		
Better/the same	1	
Poorer	1.53 [0.48; 4.83]	0.381
Current sexual quality of life*		
Better/the same	1	
Poorer	1.08 [0.48; 2.46]	0.847
Perceived a very negative impact of the COVID-19 crisis on personal life		
No	1	
Yes	**3.69 [1.43;7.46]**	< 0.001****
Perceived a very negative impact of the COVID-19 crisis on professional life		
No	1	
Yes	**3.56 [1.76; 7.26]**	< 0.001****
Missed diagnosis/treatment/surgical intervention for an important health issue during the COVID-19 crisis		
No	**1**	
Yes	**2.99 [1.49;5.98]**	0.002****
Fewer medical follow-up visits***		
No	1	
Yes	**3.06 [1.43; 6.55]**	0.004****
Needed support from friends (excluding PLHIV)***		
No need/satisfied need	**1**	0.148****
Unsatisfied need (need partially satisfied/did not receive any support)	1.65 [0.84; 3.26]	
Needed support from CHW***		
No need/satisfied need	1	
Unsatisfied need (need partially satisfied/ did not receive any support)	2.83 [1.42; 5.65]	0.003****
Needed support from medical professionals***		
No need/satisfied need	1	
Unsatisfied need (need partially satisfied/ did not receive any support)	6.12 [2.57; 14.6]	< 0.001****
Felt sufficiently informed by their HIV medical team about the risk of COVID-19 infection in the context of HIV infection and treatment		
No/do not know	1	< 0.001****
Yes	**0. 23 [0.11; 0.50]**	
Forced to disclose HIV status**		
No	1	
Yes	**3. 27 [1.56; 6.88]**	0.002****
Received ART from peer CHW (new government care policy introduced)**		
No	1	0.028****
Yes	**0.28 [0.09; 0.88]**	
Received multi-month ART (new government care policy introduced)**		
No	1	0.004****
Yes	**0.35 [0.17; 0.7]**	

*Compared to before the COVID-19 crisis.

**During the COVID-19 health crisis.

***Since the COVID-19 health crisis started. ****Missing values were imputed in the most likely category to occur. Significant values are in bold.

PLHIV age and the probability of ART interruption had a linear relationship (OR 95% CI 0.96 [0.92;0.99], p = 0.029). People with a primary school education level or no schooling ([2.24 [1.14; 4.55], p = 0.04), and persons self-identifying as PWUD (3.12 [1.54; 6.31], p = 0.002) were more likely to have interrupted ART since the crisis started. Reporting a very negative impact of the crisis on one’s personal and professional lives (3.69 [1.43; 7.46], p < 0.001 and 3.56 [1.76; 7.26], p < 0.001, respectively) was significantly associated with ART interruption. Moreover, those with fewer medical follow-up visits since the start of the health crisis (3.06 [1.43; 6.55], p = 0.004) and those reporting forced disclosure of their HIV status in the same period (3.27 [1.56; 6.88], p = 0.002) were more likely to experience ART interruption. The need for support from friends (excluding other PLHIV), from peer CHW or from medical professionals, and not receiving as much support as they needed from each of these three categories were associated with ART interruption (respectively: 1.65 [0.84; 3.26], p = 0.148; 2.83 [1.42; 5.65], p = 0.003; 6.12 [2.57, 14.6], p < 0.001).

On the contrary, PLHIV who declared they used ANSS-Santé PLUS services (0.41 [0.17; 0.95], p = 0.039), those who self-identify as belonging to at least one key population (0.35 [0.11; 1.05], p = 0.068) and those who declared that they had been sufficiently informed by their HIV medical team about the risk of COVID-19 infection in the context of their HIV infection and treatment (0.23 [0.11; 0.50], p < 0.001) were less likely to experience ART interruption. This was also true for those who received their ART from peer CHW as part of the government policy guaranteeing the provision of HIV care through new modalities (i.e. adding outreach to clinic-based services) during the crisis (0.28 [0.09; 0.8], p = 0.028), and persons receiving multi-month ART ( 0.35 [0.17; 0.72], p = 0.004).

No statistically significant difference was observed for the following factors: gender, self-identifying as an MSM or an SW, area of residence, province of residence, financial situation, quality of life, family support, and receiving support from CHW involved in a community organization.

### Factors independently associated with ART interruption since the beginning of the COVID-19 health crisis in Burundi, N = 292 (Table 3)

**Table 3:** Factors independently associated with ART interruption since the beginning of the COVID-19 health crisis in Burundi, multivariable model (N = 314).

	% of ART interruption	Multivariable analysis, N = 314	
		aOR [95% CI]	P-value
Age (years) (per one unit increase)*		**0.94 [0.89; 0.99]**	0.009***
Highest level of education			
No schooling/primary	16.1	2.44 [1.06; 5.64]	0.036
Secondary/higher education	7.7	1.00	
Perceived a very negative impact of the COVID-19 crisis on personal life			
No	7.0	1.00	
Yes	21.36	**2.81[1.26; 6.26]**	0.012***
		1.00	
Felt sufficiently informed by their HIV medical team about the risk of COVID-19 infection in the context of HIV infection and treatment			
No/do not know	19.6	1.00	0.002***
Yes	5.2	**0.24 [0.01; 0.61]**	
Forced to disclose HIV status**			
No	9.1	1.00	
Yes	24.5	**3.61 [1.49; 8.74]**	0.004***
Received multi-month ART (new government care policy introduced)**			
No	17.5	1.00	0.025***
Yes	11.5	**0.40 [0.18; 0.89]**	

*We tested the quadratic effect of age; no significant effect was found (p = 0.155).

**During the COVID-19 health crisis. ***Significant values are in bold.

After adjustment for age, ART interruption since the beginning of the COVID-19 health crisis in Burundi was significantly associated with the following: (i) having no schooling or a primary school level of education (aOR 95% CI 2.44 [1.06; 5.64], p = 0.036); (ii) being forced to disclose one’s HIV status during the COVID-19 crisis (3.61 [1.49; 8.74], p = 0.004), and (iii) a very negative impact of the crisis on one’s professional life (2.81 [1.26; 6.26], p = 0.012).

In contrast, perceiving to have been sufficiently informed by one’s HIV medical team about the risk of COVID-19 infection in the context of HIV infection and treatment and having received multi-month ART during the crisis was inversely associated with ART interruption (0.24[0.01;0.61], p = 0.002 and 0.40 [0.18; 0.89], p = 0.025 respectively).

### Reasons for interrupting ART, n = 37 (Fig. 1)

**Figure 1:**
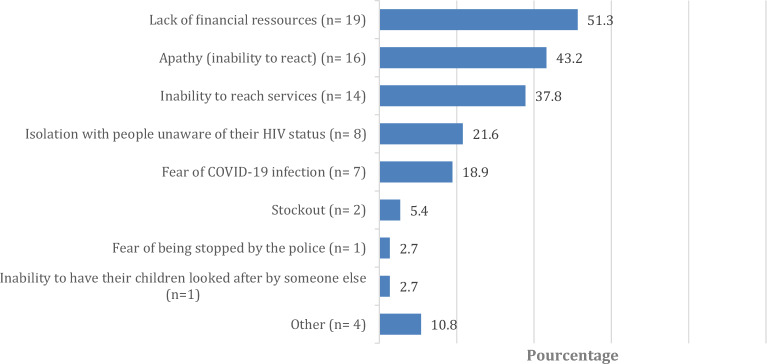
Reasons for interrupting ART* (%), n = 37. *Multiple choices possible.

Among the 37 PLHIV respondents who reported ART interruption since the beginning of the COVID-19 health crisis, the most frequently reported reason for interrupting ART was a lack of financial resources (n = 19, 51.3%). Participants who mentioned this reason latter also reported a strong deterioration of their financial situation since the beginning of the COVID-19 crisis. The second and third most-frequently reported reasons for interrupting ART were apathy (inability to react, inability to make a decision) (n = 16, 43.2%), and the inability to reach HIV health services (n = 14, 37.8%). Other reported reasons included isolating with people who were unaware of the participant’s HIV status (n = 8, 21.6%) and fear of COVID-19 infection (n = 7, 18.9%).

## Discussion

Estimating the extent to which PLHIV in Burundi interrupted ART during the COVID-19 health crisis may provide a greater understanding of the impact of crises (political, social, economic or health^8^) on HIV care in the country. It may also help to highlight possible solutions to prevent ART interruption during future crises.

In the present study, 11.7% of participants reported ART interruption during the first year of the COVID-19 pandemic in Burundi. In 2020, loss to follow-up in Burundi was estimated at 6.5% two years after ART initiation^29^. The reasons for ART interruption given by the participants in our study reflect the factors highlighted in the multivariable analysis.

The inability to reach HIV health services was one of the main reasons reported for interrupting ART in our study.. Between January and July 2021, the costs arising from obligatory face masks for use in public transportation and in places where people gathered^20,21^, most probably led people to restrict their movements^30^ and therefore limited HIV healthcare service access.

Multi-month dispensing of ART in HIV clinics was recommended in Burundi in January 2020 and implemented in July 2020 following the country’s official declaration of the COVID-19 health crisis as a way to tackle insufficient ART uptake^31^. ANSS-Santé PLUS followed this recommendation by providing three-month (instead of one-month) ART supplies to those who preferred to collect their medications at the CBO’s premises^32^. Our study found that multi-month dispensation was a positive innovation, as it was inversely associated with ART interruption. This finding reflects other studies which found that multi-month dispensing of ART acted as a facilitator to ART adherence in times of crises, especially when physical accessibility to HIV clinics was problematic^33–35^. The present study therefore suggests that in times of crises greater emphasis should be made on ensuring that PLHIV are provided with enough ART for several months, in case they are not able to go to their HIV health clinic.

Another innovation implemented in Burundi during the COVID-19 crisis was community-based distribution of ART by CHW^17,21^ to ensure continuity of care to stable PLHIV. Starting in 2021, ANSS-Santé PLUS ensured distribution through its different community delivery points^36^*.* However, our multivariable analysis did not highlight any significant difference between PLHIV who received ART from peer CHW and those who received it directly at the clinic. This result contrasts with findings in certain other contexts where community-based delivery was associated with better treatment adherence^16,35,37,38^ whether in times of crises or not^39,40^. This discrepancy may be explained by a fear of being recognized – and therefore stigmatized—as a PLHIV by persons in the general community if one were to go to an ART delivery point, something which has been observed in other contexts (e.g. Uganda)^16,38,41,42^.

In our study, the fear of COVID-19 infection was also one of the main reasons for ART interruption during the crisis. This result reflects findings in other settings where avoidance of healthcare during the crisis was associated with the same fear of exposure to the disease^30,43–46^. In Burundi, PLHIV collect their ART medications either in public or private health centers, or in community-based health facilities such as ANSS-Santé PLUS. These facilities also provide prevention services. During the COVID-19 pandemic, protective measures against infection were reinforced in health facilities^20^, including ANSS-Santé PLUS’s premises^32^. Hygiene kits were also distributed free of charge to PLHIV to facilitate their access to both public transportation and HIV health centers^32^. This paradox between the introduction of institutional preventive measures and help to access services and our respondents’ reported fear of exposure may be explained by misinformation on the risks of exposure. Our multivariable analysis highlighted that a perceived lack of information by the medical team on the risk of infection was significantly associated with ART interruption. This echoes findings in the literature where misinformation on the COVID-19 disease^47^ or on safety in health facilities^30^ led people to mistakenly avoid healthcare settings**.** Our result therefore confirms that it is essential to enhance communication between health staff and PLHIV in times of crises^48^, as well as psychosocial follow-up and therapeutic education on the importance of ART adherence^48^.

We also found that isolation with people unaware of the patient’s HIV seropositivity was problematic for ART adherence during the COVID-19 health crisis, reflecting findings in China^49^. Moreover, our multivariable analysis revealed that ART was interrupted by persons who had been forced to disclose their HIV serostatus. This reflects findings from Canada in our multi-country EPIC study^50^. Although prior work demonstrated that disclosure of one’s HIV status fosters ART adherence^51,52^, it must be done voluntarily and gradually, especially in times of crisis; PLHIV must be empowered to decide when, where and to whom they disclose their status^51^.

We found that the main reason reported for interrupting ART was a lack of financial resources due to the COVID-19 health crisis. However, in the multivariable analysis, ART interruption was not associated with a deterioration in one’s financial situation. This may be due to the fact that those people who reported a lack of financial resources as a reason for ART interruption also declared at least one other reason (e.g. difficulty to reach HIV services, fear of exposure to COVID-19 infection, isolating with people unware of their HIV seropositivity, and hunger). Many of these reasons were also identified in the multivariable analysis as associated factors. The literature shows that low household income is usually linked to multiple barriers that can compromise ART adherence. These barriers include food insecurity and high transportation fees for attending health facilities^53^. Removing these barriers would therefore mitigate the impact of low resources on ART adherence. In addition to implementing multi-month ART dispensing, ANSS-Santé PLUS provided nutritional support to PLHIV who declared a loss of income due to the COVID-19 crisis^54^. These two strategies may explain the absence in our analysis of any association between a lack of financial resources and ART interruption. Our study findings highlight the need to implement coping strategies during crises that indirectly tackle the issue of loss of income, something already proposed in other settings^17,55^.

The univariate analysis indicated that key populations were less likely to interrupt ART, although this variable was not significant in the multivariable model. In terms of the three pillars of the HIV care cascade (i.e. screening, treatment initiation, and treatment adherence), although key populations in Burundi are at greater risk in terms of new acquisition of HIV infection^5–7^, our study showed that they were not at particular risk of interrupting their ART in times of crisis. This echoes previous findings in Brazil, India and Thailand where ART intake in PLHIV identifying with key populations remained optimal (i.e. same adherence probability as that of the general population) during the COVID-19 crisis^56–58^. Our result may be due to the fact that in the ANSS-Santé PLUS organization, PLHIV identifying with key populations have long benefited – that is to say even before the COVID-19 crisis – from close monitoring by service providers and peer navigators. This includes home visits or phone calls when a PLHIV misses a medical visit or does not come to collect his/her ART supply, as well as frequent focus groups on diverse themes.

Finally, all the factors associated with ART interruption in our study were directly related to the COVID-19 health crisis. We can therefore hypothesize that this crisis played a consequential role in the amplification of ART interruption in Burundi. This is confirmed by the ANSS-Santé PLUS’ data showing that the loss-to-follow-up rate was higher during the COVID-19 crisis than in the pre- or post- COVID-19 periods^36,54,59–61^.

We remind the reader of the important strategies adopted to mitigate ART interruption in Burundi during this period. These same strategies could be used in future crises.

### Strengths and limitations

The study has several limitations. First, it only took into consideration the perceptions of PLHIV participants who physically attended the three ANSS-Santé PLUS facilities involved at the time of data collection. Therefore, all other PLHIV, including those stranded internationally are not represented. Indeed, between January and July 2021, international borders in the country were closed^20,21^. Accordingly, the true percentage of ART interruption during the crisis may have been higher than our results.

Second, the ANSS-Santé PLUS network represents 8% of PLHIV in Burundi and mainly operates in urban areas^60^. The vast majority of PLHIV in Burundi (71.6%) live in rural areas^62^. Accordingly, our study sample cannot be considered representative of the HIV epidemic profile in Burundi as a whole. Nevertheless, the longer distances to health facilities and problems with transportation in rural areas would suggest that the rate of ART interruption at the national level was even higher than that which we found. Moreover, unlike the majority of other care structures in Burundi, ANSS-Santé PLUS comprises a multidisciplinary team including healthcare workers and peer CHW. Previous findings have shown that peer CHW enhance the quality of HIV care^63^, which would suggest greater adherence to treatment. This is another reason for suspecting that the true percentage of ART interruption at the national level was higher than that found in our study.

Third, the cross-sectional design of the study was motivated by the urgent need to collect data. Yet the inherent nature of cross-sectional studies limits causal inference between the outcome and covariates. Despite this limitation, the study provides valuable information on predictors of ART interruption in Burundi.

Fourth, due to the urgent need to collect data in the context of a health crisis, convenience sampling was used to recruit PLHIV at the health facility while those identifying with key populations were actively recruited by their peer CHW into their networks. This may have generated an over-representation of key populations in our study population. This same limitation can also be considered as a study strength as the active recruitment by peer CHW enabled us to involve in this study key populations, highly affected by the HIV pandemic^5–7^. Indeed, almost half of the respondents self-identified as MSM or SW.

Fifth, due to the emergency context, our study did not collect information on refusal to participate. Therefore, selection bias cannot be excluded.

Sixth, social desirability bias is also possible as the data were collected face to face by community health workers of the ANSS-Santé PLUS. This may have been mitigated by the fact that peer CHW are recognized as trustworthy.

Finally, the variable ‘forced to disclose HIV status’ was self-reported. We had no way of confirming whether disclosure was indeed forced or not.

## Conclusion

During the COVID-19 health crisis in Burundi, 11.7% of the PLHIV participating in the EPIC program interrupted their ART. The results of this community-based research study show that specific measures can be implemented in the future in the country to minimize the risk of ART interruption in times of crisis. These include multi-month ART dispensing and enhancing communication between PLHIV and medical teams.

Our results highlighting the relatively high percentage of self-reported forced HIV status disclosure during the COVID-19 crisis, underline the necessity for disclosure to be voluntary if ART adherence in Burundi is to be improved.

## Data Availability

The data used and analyzed during this study are not openly available due to reasons of sensitivity and are available from the corresponding authors upon reasonable request. Data are located in a controlled and secured access data storage in Coalition PLUS.
